# Carbon Ligands: From Fundamental Aspects to Applications

**DOI:** 10.3390/molecules26082132

**Published:** 2021-04-08

**Authors:** Yves Canac

**Affiliations:** LCC–CNRS, Université de Toulouse, CNRS, UPS, CEDEX 4, 31077 Toulouse, France; yves.canac@lcc-toulouse.fr

Ligand design is at the forefront of many advances in various areas of chemistry such as organometallic chemistry, functional materials, and homogeneous catalysis. Faced with these challenges, the development of ligands has long been centered on elements of group 15 (N, P), and it is only more recently that carbon ligands have proven to be valuable alternatives. The predominance of N- and P-based ligands for many years was due to their high stability, as opposed to carbon ligands, which were supposed to be highly sensitive and reactive. The isolation of the first stable carbenes, namely, a phosphino(silyl)carbene by Bertrand et al. in 1988 [1], followed a few years later by the famous *N*-heterocyclic carbenes (NHCs) by Arduengo and co-workers [2] proved thus to be a trigger in the minds of chemists, leading very quickly to remarkable advances in both fundamental and applied fields of chemistry. However, if the advent of carbenes was a decisive factor, other carbon ligands demonstrated also their potential as Lewis bases in the activation of inert bonds, coordination chemistry, and homogeneous catalysis. Indeed, one must also mention the huge contribution of Schmidbaur in the chemistry of P-ylides which brought these carbon species to light [3]. Later on, different carbon ligands were developed based on a C-*sp*^2^ coordinating carbon atom similar to NHCs or, alternatively, on a C-*sp*^3^ coordinating carbon atom similar to phosphonium ylides. In the first category, we can mention the more recently reported cyclic alkylamino carbenes (CAACs) where one of the stabilizing N-atoms adjacent to the carbenic center is substituted by a quaternary carbon atom [4], and in the second category all other onium ylides in which the positively charged heteroatom is an N, As, S, or Se atom [5] without forgetting the N-heterocyclic olefins (NHOs) where the exocyclic ylidic carbon atom is stabilized by an imidazolium moiety [6]. Following Ramirez’s pioneering report on carbodiphosphoranes [7], bis-ylide species also named carbones [8], which behave like strong σ- and π-donor carbon ligands due to the presence of two lone pairs at the central carbon atom, have also recently experienced a renewed interest in coordination chemistry with applications in catalysis [9]. While all the carbon ligands cited so far have the common feature of being globally neutral in their free state, anionic carbon ligands likewise play a fundamental role in modern chemistry. Both NHCs and ylides can be thus made anionic by deprotonation to form metalated dicarbenes and ylides which correspond to ditopic anionic NHCs [10] and yldiides [11], respectively. In these species, the introduction of a negative charge modifies the electronic properties resulting in an enhancement of the nucleophilicity with repercussions, in particular, in catalysis. Other anionic carbon ligands exhibiting alkyl, alkenyl, alkynyl, allyl, aryl, or cyclopentadienyl-based backbones differing in the hybridization state and the number of coordinating carbon atoms were also widely considered over the years from fundamental aspects to applications.

In this *Special Issue*, the majority of the different types of carbon ligands mentioned above have been gathered together. The NHCs which constitute the most developed family of neutral carbon ligands have been approached from two main directions by the different contributors, namely, from a synthetic point of view with the preparation of new carbenic structures, and for their applications either in catalysis or in biology. In the first direction, while Kunz et al. described the fascinating coordination chemistry of a strongly donor macrocyclic *C,N,C*-pincer ligand exhibiting pentamethylene tethered NHC donor ends [12], Dias et al. were interested in the design of Hg(II) complexes bearing anionic maloNHC ligands [13], and Santini et al. in the chemistry of zwitterionic imidazolium borate species [14]. Regarding catalytic applications, Grela et al. reported the preparation of a family of nitro-activated Ru(II)-based olefin metathesis catalysts while evaluating the influence of the NHC ligand [15]. Huc et al. demonstrated that benzyloxycalix arene supported NHC-Pd(II) complexes were active for the Suzuki–Miyaura cross-coupling reaction in water [16], and Le Roux et al. focused on the preparation of Ti(IV) and Hf(IV) complexes containing tridentate bis-phenolate NHC core ligands for the copolymerization of cyclohexene oxide with CO_2_ [17]. For biological purposes, Bellemin-Laponnaz et al. synthetized a series of Pt(IV) NHC complexes and studied their cytotoxicity against different cancer cell lines [18], and Gornitzka et al. prepared Au(I) NHC-artemether complexes to assess their antiplasmodial activity and cytotoxicity against mammalian cells [19]. Finally, in this category of s*p*^2^-carbon ligands, the chemistry of NHC core pincer ligands bearing two anionic coordinating ends, especially phosphonium ylide moieties, was reviewed by us [20].

As a formal link between the two categories of neutral carbon ligands of interest, Gessner et al. reported a joint experimental and theoretical study on cyclic amino(ylide) carbenes based on pyrrole and trialkyl onium fragments where the carbenic center is expected to be stabilized by the strongly donating ylide substituent [21]. Bis-ylides were also considered, on the one hand by Sundermeyer et al. through the preparation of photoluminescent Cu(I) complexes of *P,C,P*-carbodiphosphorane-based ligands [22], and on the other hand by Maerten et al. with the coordinating behavior of phosphine-sulfoxide substituted carbones towards dichlorogermylene [23]. Those carbones, as well as carbido complexes featuring a naked carbon atom, were reviewed by Frenking et al. [24]. In the same family of carbon species characterized by the presence of a *sp*^3^-carbon atom, selenonium ylides were described from synthetic and structural aspects to synthetic applications by Drabowicz et al. [25]. Anionic carbon ligands were also of primary interest in this issue, as illustrated with the report by Grützmacher et al. of alkaline metal salts based on trop (C_5_H_11_) and dbcot (C_16_H_12_) moieties and the characterization of a d^8^-Rh(I) complex of the anionic trop ligand [26].
